# The first reported case of the rare mitochondrial haplotype H4a1 in ancient Egypt

**DOI:** 10.1038/s41598-020-74114-9

**Published:** 2020-10-12

**Authors:** Konstantina Drosou, Thomas C. Collin, Peter J. Freeman, Robert Loynes, Tony Freemont

**Affiliations:** 1 KNH Centre for Biomedical Egyptology, Division of Cell Matrix Biology and Regenerative Medicine, University of Manchester, Manchester, M13 9PG UK; 2 Manchester Institute of Biotechnology, University of Manchester, Manchester, M1 7DN UK; 3 School of Medicine, University College Dublin, Dublin 4, Ireland; 4 Division of Informatics, Imaging and Data Sciences, University of Manchester, Manchester, M19 9PG UK

**Keywords:** Evolution, Genetics

## Abstract

Takabuti, was a female who lived in ancient Egypt during the 25th Dynasty, c.660 BCE. Her mummified remains were brought to Belfast, Northern Ireland, in 1834 and are currently displayed in the Ulster Museum. To gain insight into Takabuti’s ancestry, we used deep sampling of vertebral bone, under X-ray control, to obtain non-contaminated bone tissue from which we extracted ancient DNA (aDNA) using established protocols. We targeted the maternally inherited mitochondrial DNA (mtDNA), known to be highly informative for human ancestry, and identified 38 single nucleotide variants using next generation sequencing. The specific combination of these SNVs suggests that Takabuti belonged to mitochondrial haplogroup H4a1. Neither H4 nor H4a1 have been reported in ancient Egyptian samples, prior to this study. The modern distribution of H4a1 is rare and sporadic and has been identified in areas including the Canary Islands, southern Iberia and the Lebanon. H4a1 has also been reported in ancient samples from Bell Beaker and Unetice contexts in Germany, as well as Bronze Age Bulgaria. We believe that this is an important finding because first, it adds to the depth of knowledge about the distribution of the H4a1 haplogroup in existing mtDNA, thus creating a baseline for future occurrences of this haplogroup in ancient Egyptian remains. Second, it is of great importance for archaeological sciences, since a predominantly European haplogroup has been identified in an Egyptian individual in Southern Egypt, prior to the Roman and Greek influx (332BCE).

## Introduction

Takabuti lived in Thebes, Egypt, during the 25th Dynasty (c. 660 BCE). Her mummified remains and the coffin containing them were brought to Belfast, Northern Ireland, in 1834 and are currently displayed in the Ulster Museum (https://www.nmni.com/our-museums/ulster-museum/Things-to-see/Takabuti-the-ancient-Egyptian-mummy.aspx ). Translation of the inscriptions on her coffin identify her as the daughter of Nespare, a priest of Amun at Thebes, and his wife Taseniret.

Takabuti’s initial morphological examination in 1834 revealed a female of 1.55 m (5 feet 1 inch) with exceptionally well-preserved dentition from which an age at death of 25–30 years was estimated. The decayed bandages, which had likely been dipped in resin, had covered the entire body including the head, arms, legs and feet (Konstantina Drosou, personal communication, December 15, 2018).

Four days after examination, the body was rewrapped in Irish linen, leaving the head, one arm and one foot exposed. More comprehensive studies of the mummy, carried out by X-ray imaging in 1987 and computerized tomography (CT) scanning in 2006, confirmed the age of 25–30 years at time of death and revealed a healthy individual with no evidence of serious childhood illness.

Although mitochondrial DNA (mtDNA) sequencing can be used to study population affinities of archaeological human remains^1^, DNA degradation in the Egyptian climate once limited the usefulness of this approach on mummified individuals^2^. Next generation sequencing, preceded by targeted hybridization capture can be used to enrich for particular components of the human genome^3^. Such techniques, have changed how DNA data can be obtained, providing rich analytical data from which sex^4^ , kinship^5^ and population affinity^6^ can be inferred. Using established hybridization capture methods and next generation sequencing we identified the mitochondrial haplotype of Takabuti and compare her haplogroup with the mitochondrial haplogroups previously identified in ancient Egyptian mummies.

## Results

**Figure 1:**
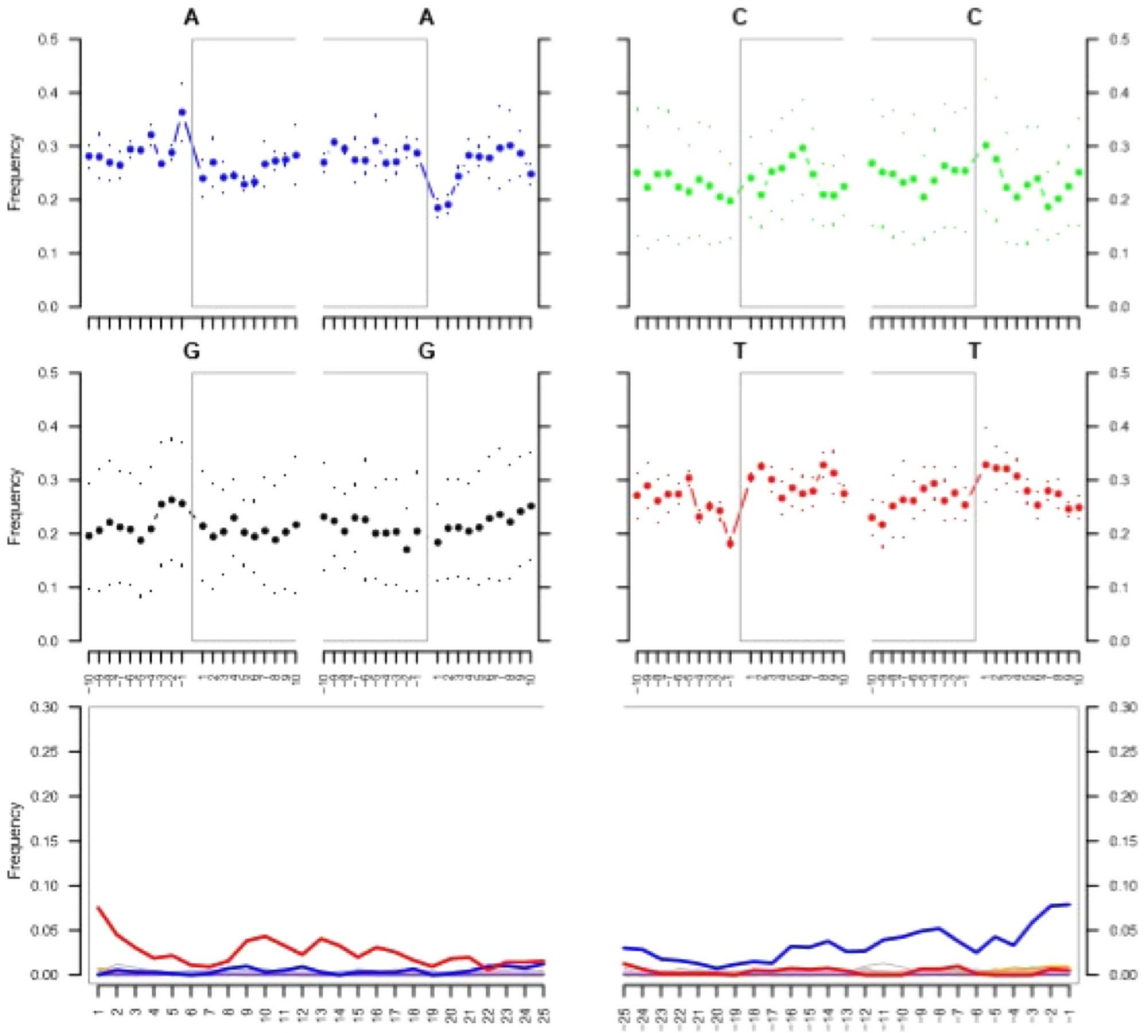
Fragmentation and misincorporation patterns for the dual enriched sequence read dataset: Data generated with MapDamage v.2.0. The upper four panels show the frequency of each of the four nucleotides at positions –10 to + 10 relative to the 5´and 3´ends of the sequence reads, and the lower two panels show the C > T (red) and G > A (blue) misincorporation patterns within the initial 25 and final 25 nucleotides of each read. The patterns are consistent with the fragmentation and misincorporation models typical for ancient DNA.

The three bone biopsies were combined in one tube, and generated bone powder of 50 mg in total. Sample was then subjected to DNA extraction and library preparation followed by two rounds of in-solution hybridization capture directed at the mtDNA (NC_012920.1) and sequenced using Illumina technology. The library prepared after the first round of capture gave 18,903,330 sequence reads, of which 81,384 were initially mapped to the mtDNA (Table 1). After removal of duplicates, this mapped dataset was reduced to 991 reads with a mean coverage of 2.9×. After the second round of capture, 24,085,550 reads were obtained, of which 2,532,752 were initially mapped to the mtDNA and 3550, with a mean coverage of 9.8× were retained after duplicate removal. In order to increase the mapping stringency, the 3550 unique reads obtained from the second capture were individually tested against the mtDNA (NC_012920.1) by BLAST analysis. This left 3001 high-confidence reads, with a mean coverage of 9.8× and maximum coverage of 131×. Analysis of the read datasets with MapDamage revealed miscoding lesion distribution patterns typical of ancient DNA (Fig. 1).

**Table 1:** Results of next-generation sequencing.

Sequencing library	Total reads	Reads mapping to mtDNA	Mapped reads after duplicate removal	Mean coverage
First capture	18,903,330	81,384	991	2.9×
Second capture	24,085,550	2,532,752	3550	9.8×

From the 3001 sequence reads from the second round of capture, 38 SNVs were identified with search parameters set at minimum variant frequency of 100% and a minimum SNV coverage of 4× (Table 2). Five of these SNVs were also identified in the read dataset from the first capture. Of these 38 SNVs, five (NC_012920.1:m.1057G > A, m.2647G > A, m.3503C > T, m.12450C > T and m.14365C > T) were considered low confidence due to their location close the 5´ or 3´ termini of sequence reads, which means that they could potentially be miscoding lesions caused by ancient DNA degradation^7^. All of the SNVs were transitions with the exception of NC_012920.1:m.6088C > A and m.6597C > G. SNVs showed no matches to personnel’s mitochondrial profiles.

**Table 2:** Summary of SNVs.

Position	Variation	First capture	*p *value	Second capture	*p *value
		Coverage		Coverage	
225	G > A	–		9×	1.0E−27
263	A > G	–		4×	1.0E−12
275	G > T	–		4×	1.0E−12
750	A > G	–		4×	1.0E−12
1057*	G > A	–		5×	1.0E−15
2647*	G > A	–		6×	1.0E−18
3370	C > T	–		7×	1.0E−21
3503*	C > T	–		4×	1.0E−12
3992	C > T	4×	5.0E−10	14×	1.0E−42
4024	A > G	4×	1.6E−11	14×	1.0E−42
4769	A > G	–		4×	1.0E−12
4964	C > T	–		7×	1.0E−21
5004	T > C	–		5×	1.0E−15
5280	A > G	–		5×	1.0E−15
6088	C > A	–		11×	1.0E−30
6360	G > A	–		7×	1.0E−21
6586	C > T	–		7×	1.0E−21
6597	C > G	–		7×	1.0E−21
6853	G > A	–		4×	1.0E−12
8860	A > G	9×	6.3E−26	15×	1.0E−45
9123	G > A	6×	2.5E−22	16×	1.0E−48
10,223	C > T	–		4×	1.0E−12
11,614	C > T	–		5×	1.0E−15
11,743	C > T	–		7×	1.0E−21
11,857	C > T	–		5×	1.0E−15
11,888	G > A	–		5×	1.0E−15
12,191	C > T	–		9×	1.0E−27
12,318	G > A	–		17×	1.0E−51
12,450*	C > T	–		6×	1.0E−18
12,697	C > T	–		8×	1.0E−24
12,709	C > T	–		8×	1.0E−24
13,274	T > C	–		27×	1.0E−81
14,274	A > G	–		5×	1.0E−15
14,365*	C > T	–		7×	1.0E−21
15,326	A > G	13×	1.3E−43	27×	1.0E−81
15,683	C > T	–		7×	1.0E−21
15,912	C > T	–		6×	1.0E−18
16,558	G > A	–		6×	1.0E−18

*Low confidence SNVs due to location close to the 5´ or 3´ termini of sequence reads. Positions in the context of reference sequence NC_012920.1.

**Figure 2:**
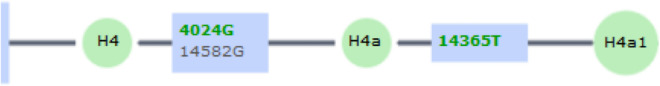
Haplogroup assignment based on Haplogrep2.

Examination of the SNVs indicated that Takabuti belonged to mtDNA haplogroup H4a1 (Fig. 2) with 90.57% confidence (Overall quality 0.906, HG Quality 1.000, Sample Quality 0.811). This assignment is based on the presence of eleven variants in total including three basal H4 variants (m.3992C > T, m.5004 T > C, m.9123G > A), along with m.4024A > G which characterizes H4a, and the low confidence SNV m.14365C > T which is associated with H4a1^8–10^. An additional SNV, m.14582A > G, which is characteristic of H4a was present in the dataset but does not appear in Table 2 as the read coverage after second capture was only 2×. Of the remaining SNVs, m.3992C > T, m.4024A > G, m.4769A > G, m.8860A > G and m.9123G > A have been found before in mtDNA variants that have been assigned to haplogroup H4a1^11–13^. The remaining of the SNVs were not included in data interpretation as they have not been described before.

## Discussion

We determined Takabuti’s mtDNA haplogroup from multiple overlapping sequence reads obtained after two rounds of hybridization capture directed at the mitochondrial genome. The sequences displayed damage patterns typical of ancient DNA, and the SNVs used to deduce the haplogroup were absent from the mtDNAs of all individuals involved in the DNA extraction and processing. Furthermore stringent steps were taken throughout all stages of this research to limit environmental and cross-contamination events (deep tissue sampling, PPE). This work was performed on bone tissue removed by minimally invasive techniques necessitating of only milligram quantities of bone and which minimised contamination. We therefore have confidence that the haplogroup we report for Takabuti is correct and not affected by modern contamination.

When interpreting our findings within the broader genetic record, the H super-haplogroup is the most common mtDNA lineage in Europe and is found also in parts of present-day Africa and western Asia^9,14^. The H4a1 variant possessed by Takabuti is relatively rare with a modern distribution including ~ 2% of a southern Iberian population^15^ , ~ 1% in a Lebanese population^12^ and ~ 1.5% of multiple Canary Island populations^13^.

Until our work neither H4 nor H4a1 has been reported in ancient Egyptian samples. However, in the archaeological record H4a1 has been reported in sixth–fourteenth century CE remains sourced from the Canary Islands, and three additional ancient DNA samples, two from Bell Beaker and Unetice contexts (2500–1575 BCE) at Quedlinburg and Eulau, both in Saxony-Anhalt, Germany^10^ , and one individual from early Bronze Age Bulgaria^15^ illustrating both the rare occurrence and sporadic distribution of this haplogroup.

**Figure 3:**
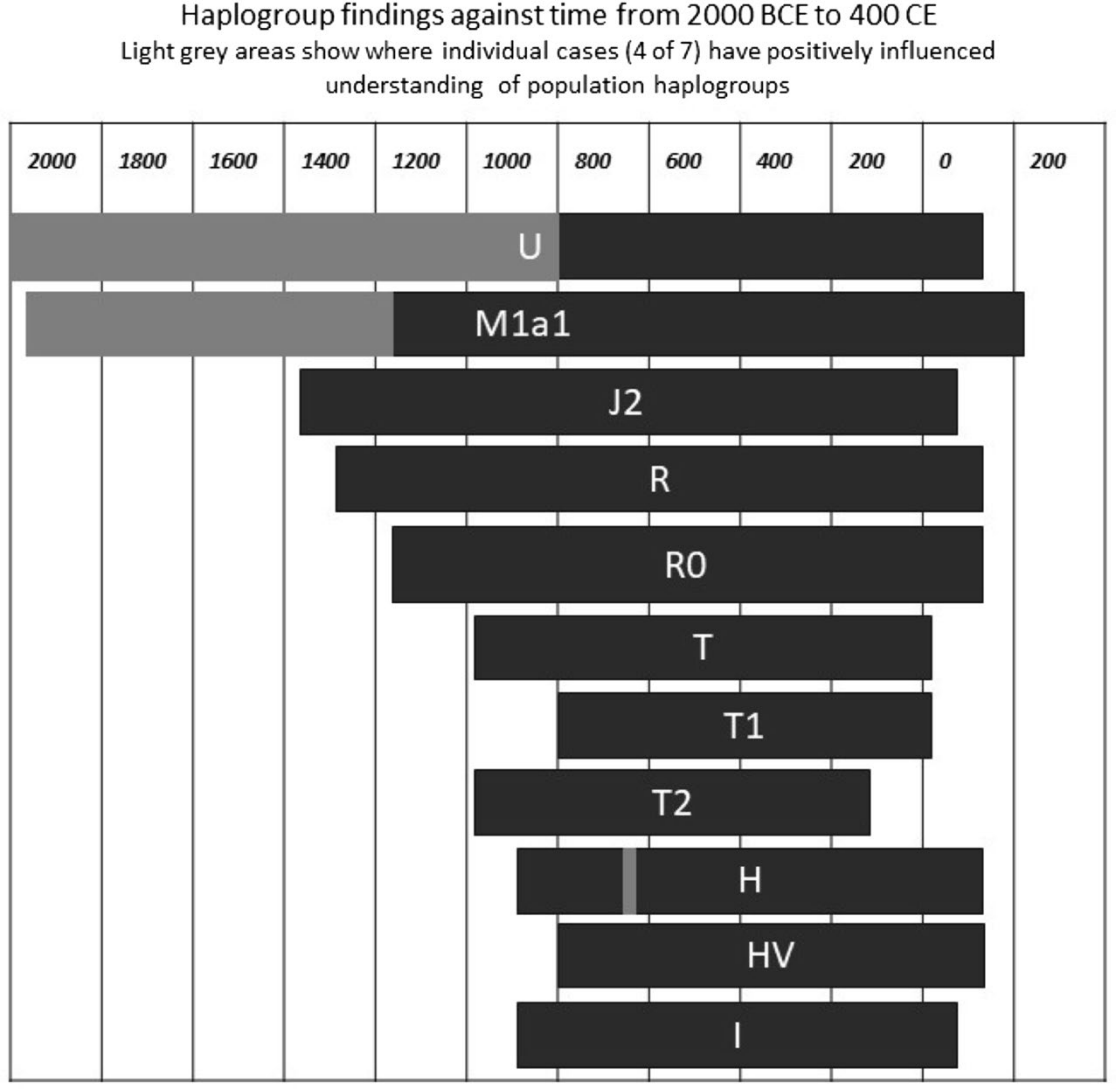
Mitochondrial haplotypes in ancient Egypt through time, from 2000BCE to 400CE. Light grey areas show where individual cases (4 out of 7) have influenced understanding of mitochondrial haplogroups. Copyright: Tony Freemont.

The overall perspective from an examination of 97 samples from ancient Egypt with a mitochondrial haplogroup (Fig. 3) is of a complicated society with a rich mixture of established genetic backgrounds, indicative of a population shaped over time by migration into the region. This is perhaps not surprising since Egypt is situated at the only land gateway between Africa and the Middle East, a region known to have been populated through the centuries by nomadic tribes and rich trading routes. As the record currently stands these are represented by the U and M1a1 haplogroups throughout the first and second millennia BCE, expanded by J2a, R0, T1, T2, HV and I in the first millennium BCE^4–6,16,17^. Superimposed on this are individuals like Takabuti with rare haplogroups, which have not been previously identified in the background.

Perhaps the most intriguing aspect of our findings, which is of great archaeological interest and importance, is the observation of a predominantly European haplogroup in an Egyptian individual located in Southern Egypt. What is fascinating is that the individual pre-dates the Roman and Greek influx (332BCE). At face value, the current genetic evidence suggests a high degree of isolation from migration into Southern Egypt. However, this finding challenges that assertion, suggesting that further investigative work could be carried out to gain a better understanding of the genetic makeup of ancient Southern Egypt.

The simplified representation of mitochondrial haplogroups in ancient Egypt in Fig. 3 demonstrates the importance of studying individuals, in order to strengthen the archaeological maternal genetic record of ancient Egypt. This extends beyond our key finding of an individual who is clearly not characteristic of the background maternal lineages based on the currently known haplotyped population. For example, in one of our previous papers we identified the M1a1 haplogroup in two mummies^5^, pushing back the earliest observation of this haplogroup in Egyptian mummies by 500 years. Similarly, a study by Loreille et al., 2018^4^ pushed back the recorded chronology of the U haplogroup in ancient Egypt by almost 1000 years. Therefore, single-case studies add to existing knowledge in this field, challenging and updating our current understanding.

Our results add to the growing body of reports demonstrating the utility of hybridization capture as a means of obtaining authentic ancient DNA sequences from Egyptian mummies^4–6^ and the importance of those findings to better understand and interpret ancient Egyptian populations.

## Methods

### Deep tissue sampling

**Figure 4:**
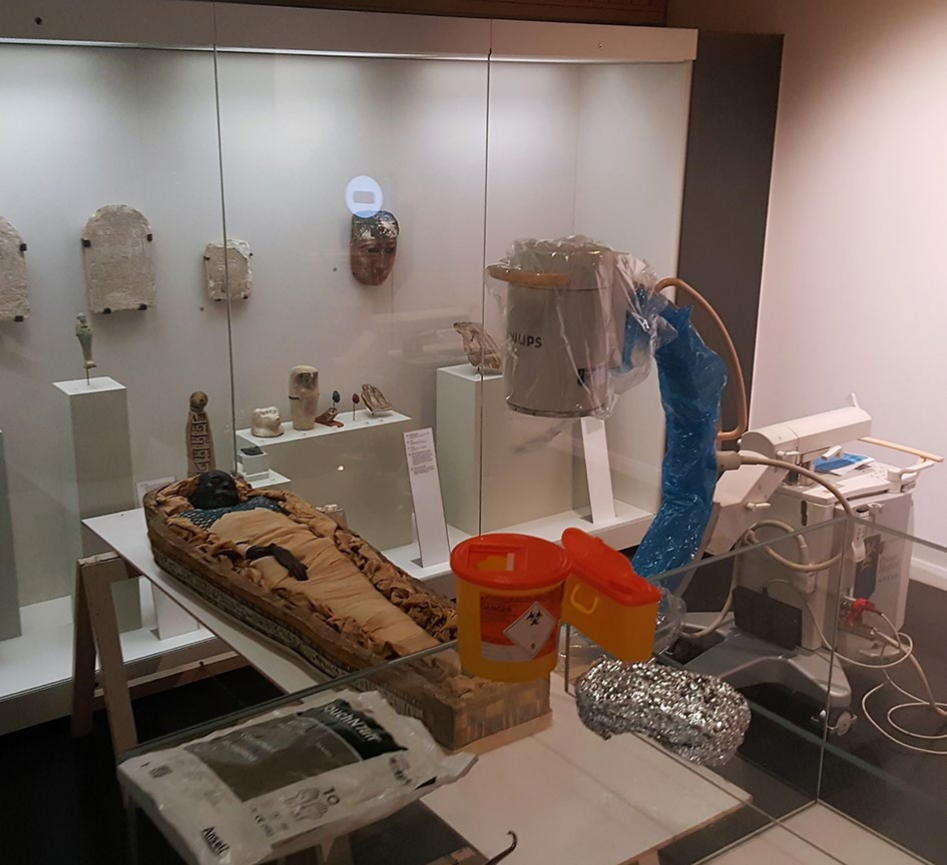
Sampling in situ. Ulster Museum, Belfast, Northern Ireland. Takabuti is positioned on the fibreboard and the X-ray imaging intensifier is positioned next to her. Copyright: Konstantina Drosou.

**Figure 5:**
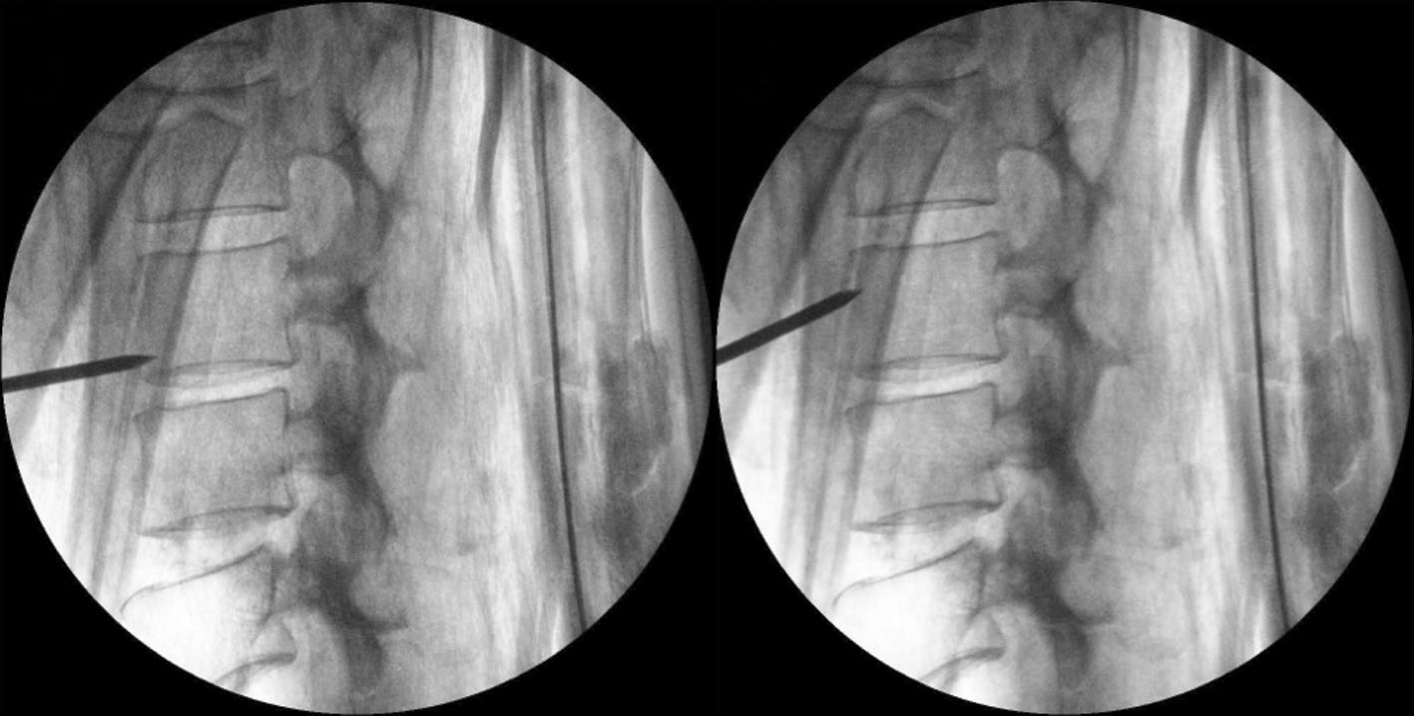
X-ray of the L3 vertebra. Biopsy needle is shown entering the body of L3 at two different angles. Copyright: Robert Loynes, Mark Regan.

Sampling was performed at the Ulster Museum in the gallery where the mummy is on display, using strict anti-contamination controls. Following isolation of the display area, the mummy was placed on a medium-density fibreboard supported by trestles that were constructed in situ for the purpose of sampling (Fig. 4). Personnel were equipped with lead jackets, forensic suits (Tyvek), boot covers, hair nets, face masks, goggles and gloves. Deep bone tissue samples (Fig. 4) were obtained with the assistance of a portable C-arm X-ray imaging intensifier, (Philips BV Endura), positioned next to the mummy to accurately locate the vertebrae, as the mummy remained wrapped to preserve her morphological integrity (Fig. 4). Three biopsies were performed in total using one biopsy needle (Murphy M2 Diamond Tip, 11 g×15 cm, UK Medical), from the lumbar vertebral body 3 (L3). The first layer of superficial bandage was retracted using a sterile surgical retractor enabling the biopsy to be performed through the deeper bandaging layer opposite the level of the L3 vertebra in the mid-line (Fig. 5). Prior to sampling, each entry point was treated with DNA-Away (Molecular BioProducts), in order to reduce contamination from dust particles. Bone powder from the biopsies was immediately transferred into three sterile 50 ml falcon tubes (one tube for each biopsy to avoid reopening) which were then wrapped in two layers of UV irradiated aluminium foil.

### Ancient DNA extraction and sequencing

Samples were transferred to the Manchester Institute of Biotechnology where DNA extraction and sequencing library preparation were performed in a set of physically isolated, restricted access laboratories, each equipped with an ultrafiltered air supply system maintaining positive displacement pressure. The laboratories were periodically sterilized by UV irradiation when not in use. All surfaces were cleaned with 5% sodium hypochlorite solution and 70% ethanol, and all utensils and equipment such as pipettes were treated with DNA-Away before and after use. Consumables such as tubes were UV irradiated (254 nm, 120,000 μJ cm^−2^ for 2 × 5 min, with 180° rotation between exposure) before use. Personal protective equipment included Tyvek forensic suits, face masks, hair nets, goggles, boot covers and two pairs of sterile gloves. DNA extraction was carried out in a Class II biological safety cabinet, and sequencing libraries and polymerase chain reaction (PCR) mixes were prepared in a laminar flow cabinet. DNA extraction was accompanied by a DNA-free negative control (normal extraction but without sample) followed by a DNA-free PCR negative control and a library preparation negative control, the latter controls set up with water rather than DNA extract^5^. To test for potential contamination during sampling and DNA processing, mouth swabs were taken from all individuals present during the tissue sampling and from individuals working in the Manchester ancient DNA labs, and these samples were anonymized and the mtDNA for each sample was typed. Samples were obtained by informed consent and all steps in this process were performed in accordance with the University of Manchester ethics regulations. Institutional governance check confirms that the work was conducted in accordance with the University of Manchester policy.

### DNA methods

DNA was extracted from 50 mg of bone powder (resulting from a total of three biopsies) following the procedure of Dabney^18^, modified by the addition of 10% (w/v) N-lauroylsarcosine to the extraction buffer and 5 M NaOAc and 5 M NaCl to the PB buffer^19,20^.

Preliminary analyses involved two overlapping PCRs^21^ directed at the mtDNA hypervariable region I (HVRI) loci (NC_012920.1:m16028-16,195 and NC_012920.1:m16210-16,340) to assess DNA preservation and endogenous DNA content, as well as a multiplex PCR using previously established primers^22^ targeting the amelogenin locus (NC_000023.11:g.11314994_11315100 (chrX) and NC_000024.10:g.6738028_6738138 (chrY)) to confirm the sex of the mummy (Table 3). PCRs were performed with the Multiplex PCR kit (Qiagen) in a final volume of 25 μl consisting of 3 μl DNA extract, 10 μM each primer and 1×Qiagen master mix. Thermocycling conditions were**:** 95 °C for 15 min; 44 cycles of 94 °C for 0.5 min, annealing temperature for 1.5 min, 72 °C for 1.0 min; 72 °C for 15 min. PCR products were examined in 1.5% and 4.0% agarose gels, purified (Qiagen MinElute Purification kit) and sequenced from both ends by the Sanger method (GATC Biotech, Cologne). Sequences were mapped to the rCRS using Geneious v8.1.8^23^. The amelogenin test failed probably due to poor preservation, whereas preliminary PCRs showed no variations.

**Table 3:** Details of PCRs.

Target	Target sequence (5′–3′)	Annealing temperature (°C)	Amplicon size (bp)	References
mtG	F-TTCATGGGGAAGCAGATTTGG R-ATGGGGAGGGGGTTTTGATGTGG	56	168	^21^
mtF	F-ACAGCAATCAACCCTCAACTATCA R-TGTGCTATGTACGGTAAATGGCTT	57	131	^21^
AMELX	F-CCCTGGGCTCTGTAAAGAATAGT R-ATCAGAGCTTAAACTGGGAAGCTG	59	106	^22^
AMELY	F-CCCTGGGCTCTGTAAAGAATAGT R-ATCAGAGCTTAAACTGGGAAGCTG	59	112	^22^

Three dual-indexed NGS libraries were prepared^24,25^ including one library and one extraction negative control. Only the sample library was enriched twice by in-solution hybridization capture (Arbor Biosciences) according to the manufacturer's instructions for degraded samples, using a baitset covering the entire mitochondrial genome. A total of 12 and 15 cycles of post-capture PCR was performed with the enriched products using the IS5 (5′-AATGATACGGCGACCACCGA-3′) and IS6 (5′-CAAGCAGAAGACGGCATACGA-3′) primers. Libraries (one enriched and two non-enriched) were quantified by qPCR (Roche LightCycler 480) and fluorimetry (Qubit 2.0), and their length distribution assessed using a bioanalyzer (Agilent). The concentration of the extraction and library controls were undetectable and therefore were not enriched or sequenced. The remaining library was then purified using the MinElute Purification kit (Qiagen) and both enrichment rounds sequenced using the MiSeq Reagent kit (v3) 2×75. Sequence data are curated at the European Nucleotide Archive under project accession number PRJEB38492.

### Data analysis

Raw data were demultiplexed using BBmap v38.07 (sourceforge.net/projects/bbmap/) and adapter sequences removed using AdapterRemoval v2.1^26^ specifying the following parameters: *trimns*, *minlength 25*, *trimqualities*, *minquality 30*, *collapse*. Reads with overlaps of at least 11 bp were collapsed into single sequences, whereas reads that did not overlap were processed separately using BEDTools v2.26.0^27^ and only confirmed forward and reversed pairs retained. Mapping was performed with BWA v.0.7.5a-r405^28^ using default *aln*, *samse* and *sampe* commands with the exception of the *l1024* parameter which is suggested for ancient DNA data. The dataset was mapped against the rCRS reference genome^29^. Subsequently, the sam files were cleaned and sorted by coordinate system and converted to bam format using default *CleanSam* and *Sortsam* commands from Picard Tools v2.18.27 (https://broadinstitute.github.io/picard). Mapped reads were extracted with Samtools v1.2^30^ using the parameters *b, q30, and F4*. Removal of duplicate sequences was performed with the script *aweSAM collapser* (https://gist.github.com/jakeenk/). Single nucleotide variations (SNVs) were called using *HaplotypeCaller* from GATK v.4^31^ and validated using Geneious v.R8^23^. The depth of SNV coverage was set at DP > 4 and only those SNVs with a 100% read coverage were retained. Mitochondrial DNA haplogroup assignment was performed using Haplogrep^32^ sand data authenticity was assessed with MapDamage v2.0^7,33^. BLAST^34^ searches were carried out online (https://blast.ncbi.nlm.nih.gov/Blast.cgi).
